# Early and Late Postoperative Tachyarrhythmias in Children and Young Adults Undergoing Congenital Heart Disease Surgery

**DOI:** 10.1007/s00246-022-03074-w

**Published:** 2022-12-14

**Authors:** Raphael Joye, Maurice Beghetti, Julie Wacker, Iliona Malaspinas, Maya Bouhabib, Angelo Polito, Alice Bordessoule, Dipen C Shah

**Affiliations:** 1 Pediatric Cardiology Unit, Department of Woman, Child, and Adolescent Medicine, Geneva University Hospital, Geneva, Switzerland; 2 Pediatric Intensive Care Unit, Department of Woman, Child, and Adolescent Medicine, Geneva University Hospital, Geneva, Switzerland; 3 Electrophysiology Unit, Cardiology Division, Geneva University Hospital, Geneva, Switzerland

**Keywords:** Atrial tachycardia, Atrial fibrillation, Junctional tachycardia, Ventricular tachycardia, Congenital heart disease, Surgery

## Abstract

The population of patients with congenital heart disease is constantly growing with an increasing number of individuals reaching adulthood. A significant proportion of these children and young adults will suffer from tachyarrhythmias due to the abnormal anatomy, the hemodynamic burden, or as a sequela of surgical treatment. Depending on the underlying mechanism, arrhythmias may arise in the early postoperative period (hours to days after surgery) or in the late postoperative period (usually years after surgery). A good understanding of the electrophysiological characteristics and pathophysiological mechanisms is therefore crucial to guide the therapeutic approach. Here, we synthesize the current state of knowledge on epidemiological features, risk factors, pathophysiological insights, electrophysiological features, and therapy regarding tachyarrhythmias in children and young adults undergoing reparative surgery for congenital heart disease. The evolution and latest data on treatment options, including pharmacological therapy, ablation procedures, device therapy decision, and thromboprophylaxis, are summarized. Finally, throughout this comprehensive review, knowledge gaps and areas for future research are also identified.

## Introduction

Arrhythmias are a widely recognized complication of cardiac surgery in both the pediatric and adult populations. Early postoperative tachyarrhythmias typically occur h to days after cardiac surgery with a reported incidence ranging between 8 and 47% [1–3], and may critically impair hemodynamic recovery. Furthermore, advances in care have led to significant improvements in survival, with more than 90% of children with congenital heart disease (CHD) in developed countries now being expected to reach adulthood [4]. In this population, the hemodynamic burden of the original cardiac malformation (hypertrophy, dilatation, and/or fibrosis) is frequently combined with the sequelae of corrective surgery such as scars and prosthetic patches. This provides an arrhythmogenic milieu for late postoperative tachyarrhythmias that can arise months to years after surgery. Overall, arrhythmias are a major cause of morbidity and mortality in children and young adults with CHD [4]. The aim of this article is to review the current literature and address management issues of most frequent early and late postoperative tachyarrhythmias in children and young adults with CHD.

## Focal Ectopic Atrial Tachycardia

### Epidemiologic Features

The term atrial tachycardia encompasses focal ectopic atrial tachycardia (FEAT) and intraatrial reentry tachycardia (IART) and atrial fibrillation (AF). In children, FEAT is typically seen in structurally normal hearts or after corrective surgery for CHD [1, 5, 6]. Postoperative early atrial tachycardia arises in about 8% of children 8 to 14 days after CHD repair [7–9]. FEAT carries the potential for significant hemodynamic compromise and is associated with a three times higher mortality rate in infants undergoing cardiac surgery [7]. Several risk factors have been identified in children and infants including neonatal age, low body weight, hypokalemia, long cardiopulmonary bypass time, inotropic support, the use of milrinone, transposition of the great arteries (TGA), total anomalous pulmonary venous return, interstage univentricular physiology, and heterotaxy syndrome [7–9].

Although less common, FEAT may occur in the late postoperative period with a reported prevalence ranging between 8 to 39% [10, 11].

### Pathophysiological Mechanisms

Early postoperative FEAT is thought to be due to rapid discharges from a non-sinoatrial focus with abnormal automaticity properties [12]. The delayed surgery to arrhythmia time suggests that the etiology isn’t limited to trauma and probably includes inflammation and anatomic substrates [7, 8].

**Fig. 1:**
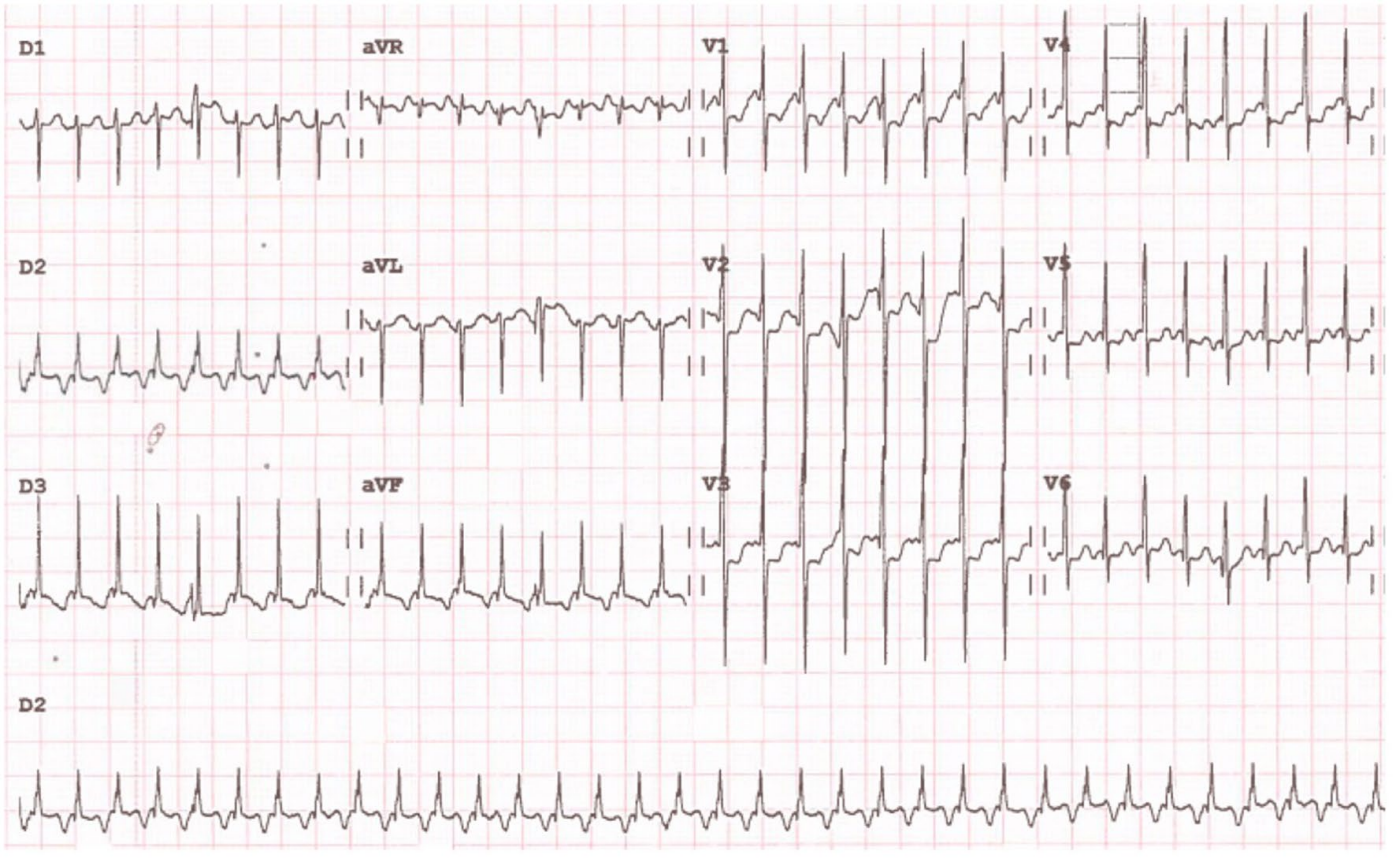
Focal ectopic atrial tachycardia in a 7-day-old boy after atrial switch for transposition of great arteries. The heart rate is around 250/min with a superior frontal P-wave axis of approximately − 90°

The electrocardiographic diagnosis criteria for FEAT (Fig. 1) include a narrow QRS complex tachycardia at a rate inappropriate for age and activity, a constant abnormal P-wave morphology, a progressive increase in atrial rate at the onset (warm-up pattern), and a variable rate depending on autonomic tone [5, 13].

Although the mechanism of late postoperative FEAT remains incompletely understood, the most likely underlying mechanism remains abnormal automaticity, or triggered activity favored by fibrosis induced intercellular decoupling with resulting greater freedom of intramyocardial potential oscillations [14]. Clinical mapping studies frequently indicate proximity to surgical scars or diseased areas and while modes of initiation and termination and response to extra-stimuli have led to suspect a microreentrant mechanism, triggered activity may demonstrate similar behavior. [11].

### Management

The therapeutic aims include control of the ventricular rate and early conversion to sinus rhythm. Yet, data on FEAT management in children with CHD remains limited. Digoxin and oral or intravenous beta-blockers showed good results in a small case–control study [9]. Clark et al. reported that rhythm control was safely achieved with monotherapy or combination of digoxin, propranolol, procainamide, and amiodarone [8]. In a retrospective review of 64 children with EAT, 57 received anti-arrhythmic therapy in the acute postoperative period; of those, 48 were still treated at hospital discharge (3 of whom were on combination therapy). Propranolol was the most widely used agent, followed by sotalol and amiodarone. Antiarrhythmic therapy was discontinued in half of the patients after several months [7]. Ivabradine inhibits the “funny current” by blocking hyperpolarization-activated cyclic nucleotide–gated transmembrane sodium channels that are up-regulated in areas of abnormal automaticity. Therefore, some authors have reported the use of ivabradine for FEAT in children with structurally normal hearts, as well as in postoperative critical patients, with satisfactory results and good hemodynamic tolerance [15, 16].

If treatable medically, catheter ablation is not recommended for atrial tachyarrhythmias that are responsive to medical treatment in the first 3 months after surgery [14]. In the case of late postoperative FEAT, targeting the site with the earliest activation relative to the P-wave was reported to be effective in adults and children [11]. More studies are needed to characterize the underlying mechanisms, the efficacy of medical agents, and to provide recommendations for catheter ablation.

## Intraatrial Reentry Tachycardia

### Epidemiological Features

IART and atrial flutter both are macroreentrant atrial tachycardias that should not be mistaken among themselves. Indeed, the reentry circuit of IART is usually caused by a central obstacle consisting of a scarring tissue or prosthetic materials, whereas atrial flutter typically rotates around the tricuspid valve annulus. [17]. IART represents the most common cause of tachyarrhythmia in young patients with CHD with a reported prevalence of 15% [18]. Its incidence increases with age and with the post-surgery time interval [14]. Several types of CHD can provide substrates for atrial tachyarrhythmias. Atrial septal defects (ASD) result in right atria volume overload and arrhythmogenic remodeling. It is probable that the longer the hemodynamic overload, the more likely the patient is to develop atrial arrhythmias. In fact, after surgical closure, IART incidence ranges from 5 to nearly 100% depending on the age at surgery and the presence of pre-procedural atrial arrhythmia [19]. Atrial switch for TGA aims at redirecting pulmonary and systemic venous return through intraatrial baffles, increasing the risk of IART to 27% [20]. Patients with univentricular physiology and Fontan circulation are also prone to atrial tachyarrhythmias. Older age at Fontan procedure, elevated right atrial pressure and cavopulmonary anastomosis have been identified as risk factors for IART [14, 21]. Finally, patients with Ebstein malformation of the tricuspid valve and tetralogy of Fallot are at high risk for postoperative atrial tachyarrhythmias, especially in the presence of a residual tricuspid regurgitation [4, 22].

IART and AF are the leading cause of morbidity and mortality in patients with CHD. Adults with CHD and atrial tachyarrhythmias have more than twice the risk of heart failure and stroke compared to younger patients with same cardiac congenital anomalies [18]. Furthermore, the yearly incidence of death in young patients with CHD and IART or AF ranges from 1 to 2% [23].

### Pathophysiological Mechanisms

Pressure and volume overload may lead to significant geometrical changes in the atria. These modifications are then associated with electrical remodeling, such as increase of P-wave duration, sinus node recovery time lengthening and conduction delay across the crista terminalis [19]. This process is combined with anatomic and electrical conduction barriers: the atrioventricular valve, right atrial free wall scars, and ASD patches were found to act as the central obstacle to reentry circuits [24]. More than one circuit may coexist in patients after ASD closure. Those referred to as double-loop or figure-of-8 reentry circuits usually involve both the atriotomy scar and the tricuspid valve simultaneously. In those patients, ablation of the cavo-tricuspid isthmus leaves a single-loop periatriotomy tachycardia, which requires additional ablation [25].

**Fig. 2:**
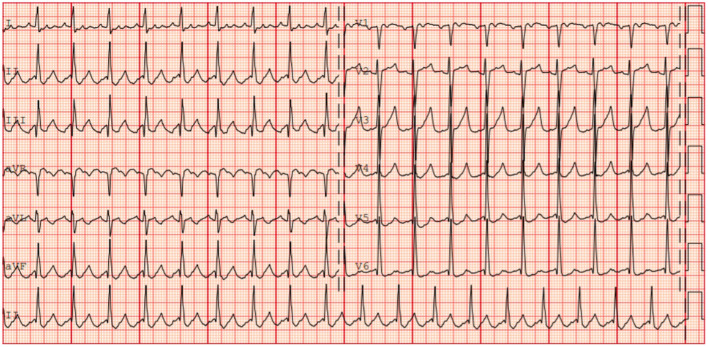
Intraatrial reentry tachycardia in a 19-year-old female after multiple surgery for aortic and mitral valves rheumatic disease. F wave is positive in the inferior leads, and negative in V1. Ventricular response is around 100/min with a 2:1 atrioventricular conduction

IART is a macroreentrant tachycardia with an atrial rate typically in the range of 150 to 250 beats per min. Although the QRS is typically narrow, both preexisting intraventricular conduction disorders and aberrant atrioventricular conduction can result in a wide QRS (Fig. 2). The P-waves may differ from the sawtooth appearance of typical atrial flutter depending on the reentrant path location [22].

### Management

Acute termination of IART or AF in patients with CHD may be achieved by synchronized electrical cardioversion, or pharmacological agents. Electrical cardioversion is recommended for hemodynamically unstable patients irrespective of arrhythmia duration or anticoagulation status [4, 14]. In patients with meso- or dextrocardia, patches or paddles should be adapted according to the location of the heart. In hemodynamically stable patients, the presence of intracardiac thrombus should be assessed by transesophageal echocardiography prior to cardioversion [4, 26].

Ibutilide, a class 3 antiarrhythmic agent, had a successful cardioversion rate of 60 to 70% in children and adults [27, 28]. Given the risk of torsades de pointes, intravenous ibutilide should be administered over 10 min in a monitored setting [4, 27]. In Europe, amiodarone is used as first-line therapy for acute termination of IART or AF due to ibutilide unavailability.

Overdrive pacing may also be considered to terminate IART in hemodynamically stable and adequately anticoagulated patients, when the arrhythmias is clearly of short duration, or an intracardiac thrombus has been ruled out [4, 14].

**Fig. 3:**
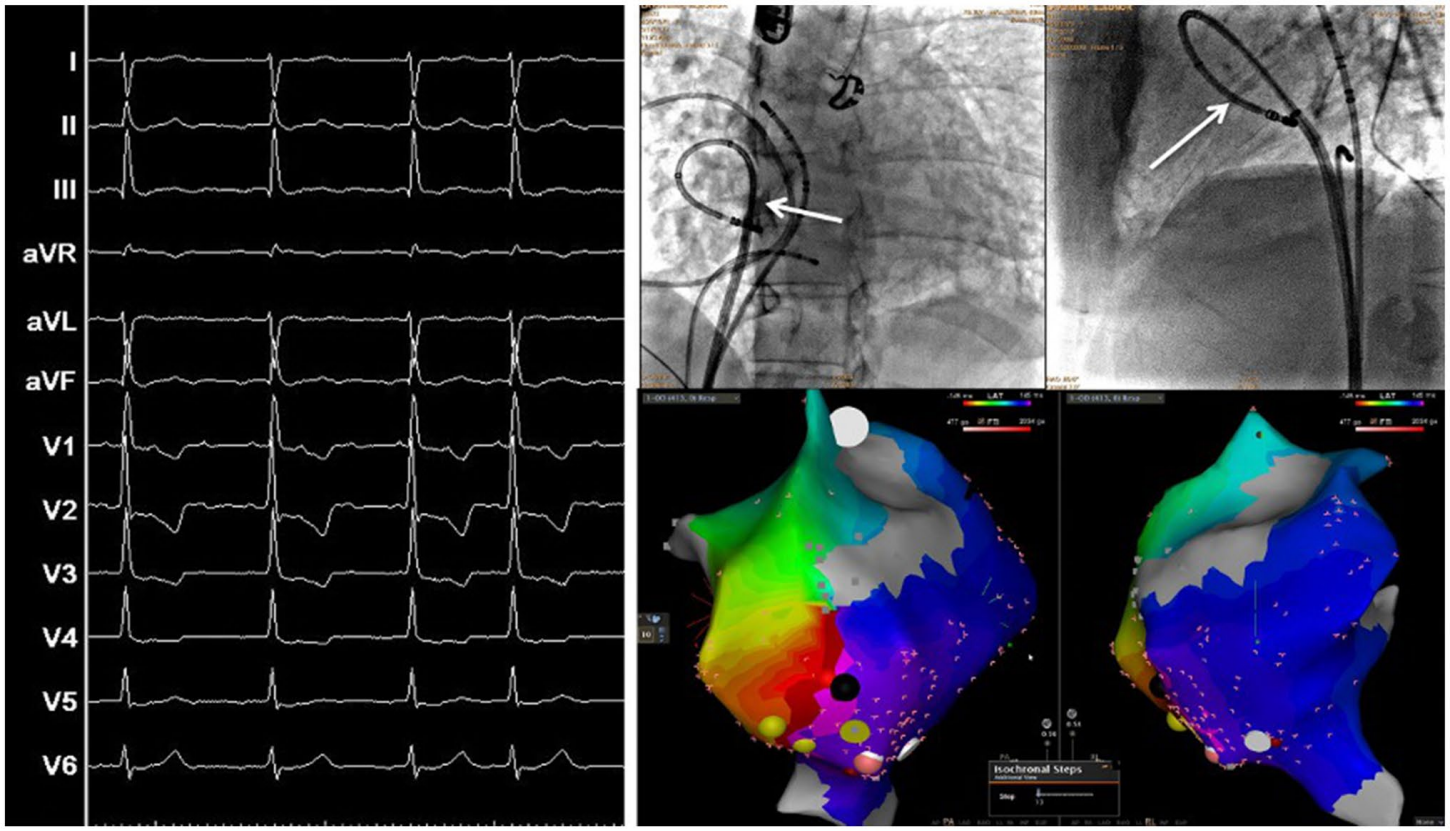
Case of a 39-year-old man with recurrent intraatrial reentry tachycardia after a Mustard procedure for d-transposition of the great arteries. The left panel shows the 12-lead ECG with a 3:1 atrioventricular conduction. The right top panel shows the fluoroscopy with anteroposterior view on the left and right latera on the left. The ablation catheter (white arrow) is in the “pulmonary venous atrium” after a trans-baffle puncture. The right bottom panel shows the activation map of the cavotricuspid isthmus (including both systemic and pulmonary venous atrial segments) in the same views as the fluoroscopy. The activation time is color coded with early activation in red and late activation in purple and shows complete conduction block of the cavotricuspid isthmus after catheter ablation with unidirectional activation going around the line of block

Long-term pharmacological management of atrial tachyarrhythmias in patients with CHD are seldom successful. A percutaneous catheter ablation approach should therefore be considered as first-line therapy in young patients with simple forms of CHD, and with amenable, circumscribed substrates (Fig. 3) [4, 14]. In patients with IART, acute ablation success rates range from 80 to 95% [14]. However, long-term recurrences are reported to occur in 15 to 50% of the cases, generally within the first year after ablation [4, 29, 30]. Consequently, multiple ablation procedures may be needed [4]. Atrioventricular nodal ablation with post-ablation ventricular pacing can be considered as third line therapy for the treatment of atrial tachyarrhythmias when other strategies have failed [14].

In patients with atrial tachyarrhythmias for whom catheter ablation is either not feasible or unsuccessful, long-term pharmacologic therapy is indicated. Rhythm control is preferred to rate control especially for moderate and complex forms of CHD [4, 14]. Atrioventricular synchrony is indeed an important determinant of hemodynamic tolerance in certain lesions such as univentricular physiology or systemic right ventricle with systolic dysfunction [4]. Class I antiarrhythmic agents are not recommended in patients with CHD and extensive ventricular scarring, coronary artery disease, and/or ventricular dysfunction [4, 14]. Class III agents have been associated with better outcomes than other antiarrhythmics in the CHD population. Given the reported increased mortality with sotalol, its use has been relegated to a class IIb indication in children and adults with preserved ventricular function [4, 31]. Amiodarone is considered the drug of choice in patients with dysfunction and/or hypertrophy of systemic ventricle [4, 14, 32]. Nonetheless, long-term administration of amiodarone is associated with significant side effects. The risk of amiodarone-associated thyroid dysfunction is indeed particularly increased in cyanotic patients and after Fontan palliation [33]. Dofetilide (not available in Europe) is considered safer than amiodarone and was associated with favorable outcomes in adults with CHD and refractory atrial tachyarrhythmias [34]. Its use is recommended as first-line therapy in patients with normal systemic ventricular function and as second line therapy in those with systemic ventricular dysfunction [4, 14]. In case of sinus rhythm conversion failure, rate control using beta-blockers or calcium channel blockers may be considered [14].

Thromboembolic complications related to atrial tachyarrhythmias are a major cause of morbidity and morbidity in the CHD population with an estimated prevalence up to 100 times higher than in controls [35]. Indeed, patients with moderate and complex form of CHD such as uncorrected cyanotic heart disease, Eisenmenger physiology, ASDs, and the Fontan circulation, present an increased risk of thromboembolic events, even in the absence of arrhythmias. [14]. As data on the safety and efficacy of non-vitamin K oral anticoagulants are limited so far, thromboprophylaxis with vitamin K antagonists is still indicated in all patients with IART or AF and moderate or complex CHD [4, 26]. For atrial arrhythmias associated with nonvalvular simple forms of CHD, the decision to start the thromboprophylaxis may be guided by established scores (e.g., CHA_2_DS_2_-VASc) [4, 14]. In this subgroup of patients, non-vitamin K oral anticoagulants seem to represent a safe alternative to vitamin K antagonists [4, 26]. Finally, anticoagulation is recommended for at least 3 weeks before and 4 weeks after cardioversion for atrial tachycardia of unknown or > 48 h’ duration in simple nonvalvular forms of CHD, and in all moderate or complex forms regardless of the arrhythmias’ duration [4].

With improved survival of the CHD population, the need for cardiac surgery has extended into adulthood. In these patients, surgical management of arrhythmias can be performed within the same operating time with only little additional risk. The maze procedures consist in creating atrial lesions in the pattern of a maze to interrupt reentry circuit without preventing normal atrial activation during sinus rhythm. In a study reporting arrhythmias operations outcomes in patients with associated CHD of various complexity (excluding Fontan procedure), freedom from arrhythmia recurrence, was 94% and 85% for atrial arrhythmias at 1 and 10 years, respectively [36]. The modified right atrial maze and the left atrial Cox-maze III have also been adapted for Fontan patients presenting with right or left atrial tachyarrhythmias [37]. Baker et al*.* described their strategy for performing the maze procedure in single ventricle patients. The freedom from recurrence of atrial reentry tachycardia at 5 years was 86%, and as high as 98% for AF [38].

## Atrial Fibrillation

### Epidemiological Features

AF is the most common type of arrhythmia in adults worldwide with a lifetime risk of 37% [39]. In patients with CHD, the risk of AF is 22 times greater and occurs at a younger age [23]. The reported incidence of AF in young patients with CHD during a mean follow-up of 27 years is around 3% [23] and increases exponentially with time, surpassing IART as the most common arrhythmia over the age of 50 [26]. Patients with CHD and the general population share the same risk factors for AF, namely age, arterial hypertension, heart failure, obesity, and diabetes [39]. In addition, left heart obstructive lesions, late ASD closure, Fontan procedures, and conotruncal defects have been associated with a high risk of late postoperative AF [14, 18, 23, 40].

### Pathophysiological Mechanisms

AF is caused by the interplay between several microscopic disarrays that take place at the atrial level following long-term atrial exposure to volume overload, including stretch-induced fatty infiltration, inflammation, ischemia, and ion-channel dysfunction [39]. As pediatric atrial myocardium is relatively resistant to hemodynamic stress. Early postoperative AF is very rare in children [41].

**Fig. 4:**
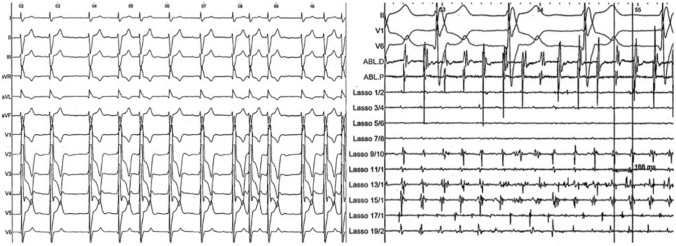
Atrial fibrillation in a 35-year-old man after a Senning procedure for d-transposition of the great arteries. The left panel shows the 12-lead electrocardiogram with irregularly irregular R-R intervals and no discernable P-wave. The right panel shows an intracardiac atrial electrogram recorded during catheter ablation. Atrial electrograms are irregular and of varying morphology and exhibit a very short cycle length (188 ms)

Electrocardiographic characteristics include irregularly irregular R-R intervals (when atrioventricular conduction is not impaired), irregular and rapid atrial activation including non-discernible fibrillatory waves (f-waves), or the absence of distinct repeating P-waves [39] (Fig. 4).

### Management

Recommendations for acute termination, long-term medical therapy, and thrombo-embolic prophylaxis are the same as for IART [4]. Rate control might be more easily achieved in AF, but rhythm control remains the first choice particularly in patients with moderate or severe CHD [4].

Before beginning long-term antiarrhythmic therapy in a child or a young adult with CHD or opting for a rate control strategy, percutaneous catheter ablation should be considered. In a heterogenous population of patients with AF and various forms of CHD, the ablation’s success rates were reported to be between 42 and 63% at one year, and around 25% at 5 years, in expert adult CHD centers [26, 42].

## Junctional Ectopic Tachycardia

### Epidemiological Features

Junctional ectopic tachycardia (JET) encompasses tachyarrhythmia originating either in the atrioventricular node or in the His bundle [43]. Postoperative JET typically arises within the first 24 h after heart surgery [44]. Its reported incidence ranges from 5 to 11% [45–48]. Young age, longer cardiopulmonary bypass and aortic cross-clamp times, low level of plasma magnesium, higher body temperature and inotropic support significantly increase the risk of postoperative JET [1, 6, 45]. From a surgical standpoint, Walsh et al*.* reported a strong association with procedures involving ventricular septal defect (VSD) closure [49]. Furthermore, Dodge-Khatami et al*.* hypothesized that tetralogy of Fallot repair was associated with a greater risk of JET that might be explained by an indirect injury to the His bundle following traction through the right atrium to relieve right ventricular outflow tract obstruction [50]. More recently, even surgical procedures thought to be less aggressive toward the atrioventricular region, such as Norwood procedure, arterial switch, total anomalous pulmonary venous return, and Fontan palliation have been identified as independent risk factors [44, 45].

### Pathophysiological Mechanisms

Even though the exact mechanism of postoperative JET remains unclear, it has been postulated that, following surgical manipulation, streaks of blood and fibrosis might infiltrate the atrioventricular conduction area resulting in abnormal automaticity [43, 49, 50]. Its onset is generally insidious with a “warm-up” pattern and the heart rate varies in accordance with the adrenergic tone. Hemodynamic compromise is often observed at higher rates due to atrioventricular asynchrony and reduction of filling time.

**Fig. 5:**
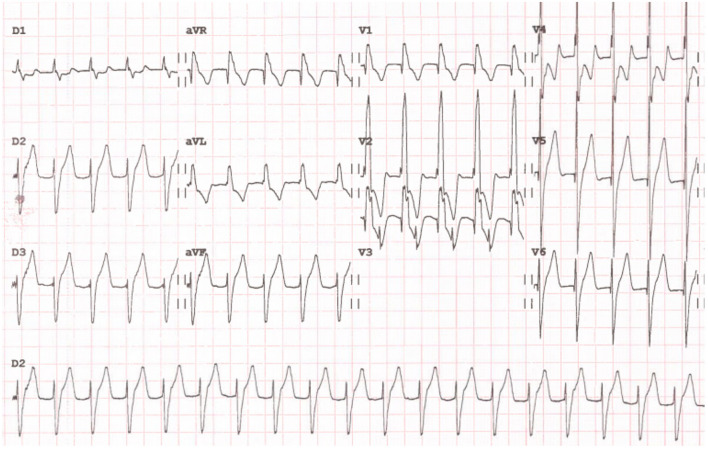
Junctional ectopic tachycardia in a 3-year-old boy after Nikaidoh procedure for correction of double outlet right ventricle, transposition of the great arteries and right ventricular outflow tract obstruction. The heart rate is around 150/min with narrow QRS complexes like that in sinus rhythm. Retrograde P-wave are seen in V3 lead using atrial pacing wire

The electrocardiographic features include QRS complexes similar to those seen in sinus rhythm and complete atrioventricular dissociation. Ventriculoatrial conduction may also be observed with retrograde P-waves [43, 48]. In cases where atrioventricular dissociation is difficult to identify, the ECG from an atrial pacing wire might be useful (Fig. 5).

### Management

As postoperative JET is a self-limited arrhythmia that usually resolves within 2 to 7 days [50], management strategies are typically aimed at ensuring good hemodynamic tolerance until sinus rhythm recovery is achieved.

General and prophylactic measures include targeting normothermia, maintaining a good level of analgesia and sedation, correcting electrolytes disturbances, and avoiding catecholamine excess. In addition, magnesium appears to have a protective effect. Several studies have suggested that the administration of 25 to 50 mg/kg of magnesium sulfate during or after rewarming during cardiopulmonary bypass may reduce the incidence of postoperative junctional tachycardia by up to 17% [51, 52]. Moreover, two prospective randomized studies showed that perioperative sedation by dexmedetomidine significantly decreased the occurrence of postoperative JET [53, 54]. An association between prophylactic use of amiodarone and a significant reduction in the incidence of postoperative JET in children was also observed [53]. However, the use of prophylactic amiodarone should be limited because of its side effects [52].

Lower body temperature is associated with a decrease of the automaticity and the conductivity of cardiac cells [55]. Therefore, therapeutic cooling is of paramount importance in the management of postoperative JET. Several pediatric studies reported the reduction of junctional rate, hemodynamic improvement, and restoration of atrioventricular synchrony with the use of therapeutic hypothermia [49, 56]. A body temperature of 32 to 34 °C can be safely obtained by external cooling or by intravenous 4 °C saline infusion. Hypothermia side effects are usually limited to shivering resulting in longer duration of sedation and mechanical ventilation. Lowering body temperature below 32 °C is not necessary nor suggested as it is associated with more severe side effects such as bradycardia, prolonged QT interval, and coagulation and immunity disorders [48, 56].

Antiarrhythmic drugs can be added in case of hemodynamically unstable or persistent JET to achieve rate or rhythm control. Procainamide, propafenone, and flecainide have been shown to effectively achieve rate or rhythm control with limited side effects [57–59]. Beta-blockers and sotalol have been used safely for rate control [48]. In the 1990s, amiodarone was introduced into postoperative pediatric cardiac care and is currently the most widely used antiarrhythmic drug for postoperative JET [52]. The administration of intravenous amiodarone was reported to be safe and effective in children with postoperative JET [60]. In 2005, the first randomized, double-blind, therapeutic trial of amiodarone in children found a significant dose response for a variety of tachyarrhythmia but failed to demonstrate independent response for different subtypes including JET. A high rate of dose-related adverse events including hypotension, bradycardia, and atrioventricular block was also reported [61]. Recently, Arvind et al. compared amiodarone with ivabradine for the management of postoperative JET in children and concluded noninferiority of oral ivabradine to intravenous amiodarone, with similar rates of conversion to sinus rhythm and an improved safety profile. Even though the use of ivabradine seems promising in this population, further data are needed to better characterize its efficacy and safety [62].

Pacing therapy at a slightly higher rate than junctional rate is an attractive solution to improve hemodynamic tolerance and should be started early in conjunction with the other measures. Atrial pacing has been shown to improve cardiac output by restoring atrioventricular synchrony [63]. The R-wave synchronized pacing, or AVT mode, senses the ventricular R-wave and triggers an atrial stimulation immediately before the following QRS complex. This attractive alternative does not increase the patient’s heart rate and thus allows a close monitoring of the arrhythmia’s natural course [64]. Finally, paired ventricular pacing might reduce the mechanical heart rate by half by implementing an artificial refractory period. However, this pacing mode is complex and requires the use of a stimulator for electrophysiologic studies [65].

Several management algorithms for JET have been proposed since the first staged treatment protocol published by Walsh et al. in the late 1990s [49]. A balanced approach should start with the implementation of general measures. Therapeutic hypothermia, antiarrhythmic medication, and pacing therapy are eventually introduced [44, 66].

## Ventricular Tachyarrhythmias

### Epidemiological Features

The ventricular arrhythmias encountered in patients with CHD encompass ventricular ectopics, nonsustained, sustained monomorphic or polymorphic ventricular tachycardias (VT) and ventricular fibrillation. Isolated premature ventricular beats usually arise early after surgery and are well tolerated unless there is constant bigeminy. Early postoperative VT is rare and commonly arises in the setting of myocardial ischemia, which makes the arterial switch and the Ross procedure particularly risky [67]. The incidence of late ventricular arrhythmias increases over time, reaching significant proportions in the adult population [3]. The clinical manifestations range from palpitations to sudden cardiac death (SCD). Ventricular arrhythmias are reported to be the leading cause of SCD in patients with CHD with an estimated overall risk 25 to 100 times greater than in an age-matched control population [68]. However, the reported incidence of sustained VT and SCD in adults with CHD remains low, ranging from 0.1 to 0.2% per year [4].

### Pathophysiological Mechanisms

The underlying mechanisms of ventricular arrhythmias vary according to the CHD type as well as and the surgical procedure and therefore have an impact on their management. Monomorphic VT is most often macroreentrant and is uncommon in the absence of a ventricular scar. Indeed, the reentry circuit includes an isthmus of slow conduction bordered by unexcitable tissue like scars or patches [69, 70]. Tetralogy of Fallot is the most common substrate for sustained monomorphic VT. As a matter of fact, four anatomic isthmuses have been identified [4]. The first is bordered by the tricuspid annulus and a right ventricular outflow tract or free wall scar/patch; the second by the pulmonary annulus and a right ventricular outflow tract or free wall scar/patch, the third by the pulmonary annulus and the perimembranous VSD patch, and the fourth by a muscular VSD patch and the tricuspid annulus [69]. Polymorphic VT and ventricular fibrillation are usually associated with more extensive myocardial injury such as hypertrophy, diffuse fibrosis, or ischemia. In CHD patients, systemic ventricular dysfunction seems to be the most important predictor of ventricular arrhythmias [70].

The electrocardiographic criteria of VT include a wide complex tachycardia with a different axis from that of sinus rhythm. Atrioventricular dissociation, fusion, and capture beats may help recognize ventricular arrhythmias. As patients with CHD often present ventricular conduction anomalies, it can be difficult to distinguish supraventricular tachycardia with aberrant conduction from VT.

### Management

Acute termination of ventricular arrhythmias in hemodynamically unstable CHD patients should be achieved according to the American Heart Association guidelines for Adults and Pediatric Advanced Life Support recommendations [71, 72]. In case of hemodynamically stable patient with monomorphic VT, pharmacological conversion can be obtained with the use of intravenous amiodarone, procainamide, or lidocaine [4]. In the early postoperative period, atrial overdrive pacing can help to control the arrhythmia while identifying and correcting reversible causes [73].

Long-term management of ventricular arrhythmias can become rapidly complex and requires a case-by-case consultative interaction between pediatric cardiologists and electrophysiologists. As in the general population, an implantable cardioverter-defibrillator (ICD) is indicated for secondary prevention of SCD in adults with CHD resuscitated from cardiac arrest and in those with spontaneous sustained VT in the absence a clear reversible cause [4]. Recommendations for the primary prevention of SCD in patients with CHD are less clearcut compared to the general population as risk stratification of patients with CHD remains an imperfect science. As a Class I recommendation, ICDs are indicated for primary prevention of SCD in adults with a biventricular physiology CHD, and a left ventricular ejection fraction ≤ 35%, and New York Heart Association (NYHA) Class II or III symptoms [4]. However, the ideal ejection fraction cut-off for primary prevention in patients with systemic right ventricles remains to be determined [74]. The relationship between inducible sustained monomorphic VT and risk of SCD remains unclear across all forms of CHD [4]. Indeed, only five subtypes of CHD account for the majority of the reported mortality, namely tetralogy of Fallot, TGA after atrial switch, congenitally corrected TGA, left ventricular outflow obstruction, and Eisenmenger syndrome [74]. In adult patients after tetralogy of Fallot repair, left ventricular diastolic dysfunction, longer QRS duration, prior palliative shunt, ventriculotomy scar, non-sustained and inducible sustained VT independently predicted appropriate ICD shocks [74, 75]. Despite the high incidence of SCD in patients after atrial switch procedure, the risk stratification remains difficult. As a matter of fact, a correlation between inducible and clinical VT has not been observed in patients with intraatrial redirection procedures. This might be explained by the fact that malignant ventricular arrythmias are often triggered by supraventricular arrhythmias in this population [76]. We believe that the therapeutic approach in this population should be individualized according to the type of cardiac lesion.

Antiarrhythmic drugs may be helpful in reducing recurrent ICD shocks, even though specific data regarding the CHD population are scarce. When it comes to antiarrhythmic drug choice, potential drugs’ side effects as well as patients’ comorbidities should be taken into consideration. Sotalol has been associated with a reduction of appropriate and inappropriate ICD discharge in patients with normal and reduced ejection fraction [77]. Amiodarone also showed satisfactory results in decreasing the risk of SCD and other cardiovascular deaths in a meta-analysis that included 15 randomized controlled trials [78]. Small series reported a significant reduction of ventricular arrhythmias with mexiletine and phenytoin after CHD repair [4]. In a European position paper, the use of beta-blockers was suggested by expert consensus for non-sustained VT to reduce the risk of tachyarrhythmia induced cardiomyopathy [14].

**Fig. 6:**
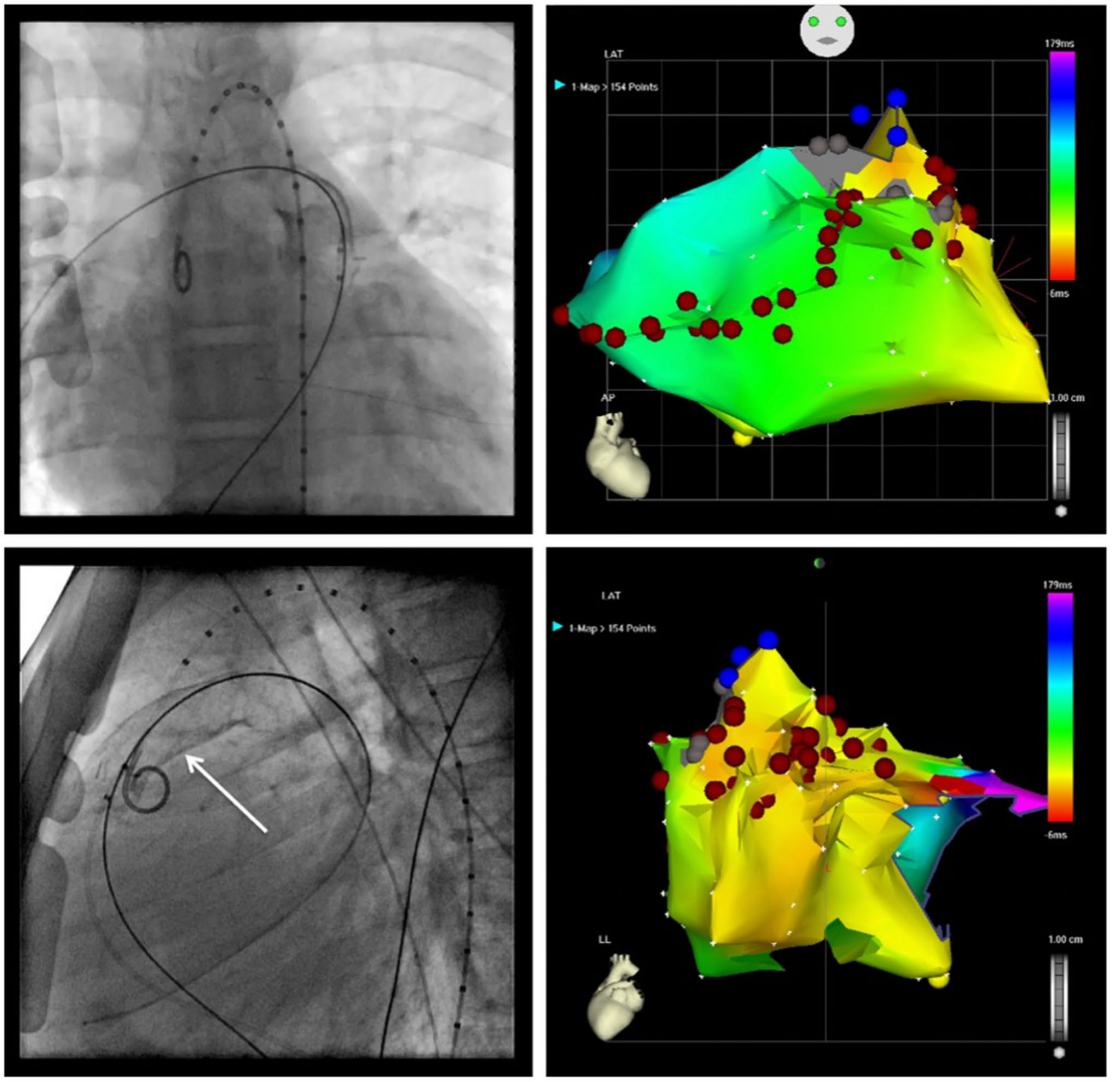
Case of a 14-year-old boy who presented recurrent episodes of monomorphic ventricular tachycardia after a Rastelli procedure for double outlet right ventricle with d-transposition of the great arteries and a large ventricular septal defect. The left panel shows the fluoroscopy with anteroposterior view on top and left lateral below. A catheter is in the right pulmonary artery via the right ventricle, and a graduated pigtail catheter is in the aorta and aortic root. The white arrow indicates the right ventricular to pulmonary artery conduit. The right panel shows an activation map of the right ventricle in the same views as the fluoroscopy. The activation time is color coded with early activation in orange and late activation in purple. Red dots indicate ablation lesions, delivered in a contiguous arrangement. The gray zone indicates scarring tissue

Finally, electrophysiologic studies including programmed stimulation might assist with risk stratification. They are usually performed to identify arrhythmia substrates that may be treated by ablation or incisional lesion [70]. Preoperative electrophysiologic studies are therefore recommended in the CHD population in case of palpitations history, unexplained syncope, and non-sustained VT [4]. Catheter ablation is indicated as adjunctive therapy to ICD in adults with CHD in case of recurrent monomorphic VT and multiple appropriate shocks not manageable by device reprogramming and/or drug therapy (Fig. 6).

Catheter ablation might also be considered for patients with frequent premature ventricular beats associated with a deteriorating ventricular function [4]. Zeppenfeld et al*.* showed that isthmuses’ boundaries can be identified with 3-dimensional substrate mapping and connected by ablation lines during sinus rhythm resulting in non-inducibility of VT in 11 adults after CHD repair [69]. These findings should facilitate catheter ablation of VT, especially in unstable patients. As for today, the identification of CHD patients with ventricular arrhythmias for whom catheter ablation might replace ICD implantation remains very challenging.

## Conclusion

This article summarizes current knowledge regarding the mechanisms and management of postoperative tachyarrhythmias in patients with CHD. Even though the outcome of patients with CHD has dramatically improved over the last decades, the occurrence of arrhythmias still holds the potential to negatively affect postoperative outcomes. Our understanding of the mechanisms underlying different types of arrhythmias and their management deserve further investigation as it might substantially contribute to improve the management of children and adults with CHD.
