# Correction: Early and Late Postoperative Tachyarrhythmias in Children and Young Adults Undergoing Congenital Heart Disease Surgery

**DOI:** 10.1007/s00246-023-03135-8

**Published:** 2023-02-27

**Authors:** Raphael Joye, Maurice Beghetti, Julie Wacker, Iliona Malaspinas, Maya Bouhabib, Angelo Polito, Alice Bordessoule, Dipen C. Shah

**Affiliations:** 1 Pediatric Cardiology Unit, Department of Woman, Child, and Adolescent Medicine, Geneva University Hospital, Geneva, Switzerland; 2 Pediatric Intensive Care Unit, Department of Woman, Child, and Adolescent Medicine, Geneva University Hospital, Geneva, Switzerland; 3 Electrophysiology Unit, Cardiology Division, Geneva University Hospital, Geneva, Switzerland

**Correction to: Pediatric Cardiology (2022) 44:312–324** 10.1007/s00246-022-03074-w

In the original publication of the article, only family names were mentioned.

The authors’ full names are:

Raphael Joye, Maurice Beghetti, Julie Wacker, Iliona Malaspinas, Maya Bouhabib, Angelo Polito, Alice Bordessoule & Dipen C. Shah.

This has been corrected in this paper.

